# Effects of the Program for Developing Oral Communication Expressiveness on university radio announcers: a pre/post intervention study

**DOI:** 10.1590/2317-1782/e20240122en

**Published:** 2025-02-10

**Authors:** Denis de Jesus Batista, Aline Santos da Conceição

**Affiliations:** 1 Programa de Pós-graduação em Modelos de Decisão e Saúde, Centro de Ciências Exatas e da Natureza, Universidade Federal da Paraíba – UFPB - João Pessoa (PB), Brasil.; 2 Dr Liang Voice Program, Faculty of Medicine and Health, The University of Sydney - Sidney, Australia.; 3 Centro Universitário Jorge Amado – Salvador (BA), Brasil.

**Keywords:** Speech, Radio, Voice Training, University, Voice

## Abstract

**Purpose:**

To analyze the effects of the Program for Developing Oral Communication Expressiveness on announcers of a university radio station.

**Methods:**

This is a pre/post intervention study. The Program for Developing Oral Communication Expressiveness was implemented with eight announcers in eight 2-hour meetings. Participants were evaluated before and after the program using self-perception instruments, auditory-perceptual judgment, and acoustic analysis of their voices during standardized readings. Statistical analysis included descriptive and statistical inference methods with a significance level of p < 0.05.

**Results:**

The announcers’ self-perceived diction and voice improved after the program. Half of the pairs evaluated improved in the auditory-perceptual judgment of reading, emphasizing voice quality, speech, and interpretation. The acoustic analysis found reduced fundamental frequency of speech, increased articulation and elocution rate, and decreased speech intensity.

**Conclusion:**

The program considerably improved aspects of the announcers' voice and speech, highlighting the importance of specific training programs to improve radio communication skills.

## INTRODUCTION

University radio stations play a fundamental role in academic, cultural, and social communication. As spaces for experimentation and content production, these stations provide university students with the opportunity to develop communication and artistic skills while also serving as platforms to inform and entertain the academic community and society at large^(1,2)^.

Oral communication competence is crucial for successful radio broadcasts, as it directly impacts the announcers’ ability to convey clear, coherent, and engaging information^(3)^. The definition of communication competence may vary depending on the context where the term is used, but it generally refers to an individual's ability to use language effectively and appropriately in different communicative contexts. This involves understanding the principles of verbal and nonverbal communication in ways that suit various situations, such as public speaking, informal conversation, and video recording for social media, among other communicative contexts^(4)^. In other words, a person with communication competence can adapt their use of language based on the context, audience, and purpose of the interaction, ensuring that their message is clear, relevant, and appropriate to the situation at hand^(5)^. The need for specific training to develop this competence is even more crucial in university radio stations, where announcers are often students still in training^(4,6)^.

The scientific literature has highlighted the importance of training to improve oral communication across various professional contexts^(7-9)^, including radio broadcasting^(4,6,10-12)^ and professional training, such as undergraduate education^(13,14)^. Studies show that interventions for this audience can have positive effects on vocal quality, diction, modulation, and other aspects of oral communication, contributing to greater awareness and competence in message delivery and interaction with the audience^(4,6-10)^.

There is an evident need for scientific research exploring the effects of oral communication development programs on university radio announcers^(15)^. An intervention study with this population could provide valuable insights into the effects of such training and help improve training and professional development practices for future occupational voice users. Thus, this study aimed to analyze the effects of the Program for Developing Oral Communication Expressiveness on university radio announcers, using a multidimensional analysis that integrates data from self-perception of voice and speech, auditory-perceptual judgment, and acoustic analysis.

## METHODS

### Study design

This is a pre/post intervention study.

### Ethical aspects

The study began after the Research Ethics Committee’s approval, according to evaluation report no. 2.780.453 of protocol CAAE 87624618.0.0000.0041, and the radio station’s consent. All study participants agreed to participate by signing an informed consent form.

### Participants

The invitation to participate in the study was extended during an administrative meeting at the radio station. The inclusion criteria were being a student and belonging to the university radio’s broadcasting team. The exclusion criteria were having cognitive deficits or difficulties in understanding and performing the proposed evaluations and exercises, being engaged in communicative or vocal training during the study period, having a recent history of dysphonia or dysphonia symptoms, being a smoker, being pregnant, or failing to participate in all research stages.

The initial sample had nine participants. However, one withdrew during the process, resulting in eight announcers.

The study involved eight announcers from the university radio station, of whom five were females, and three were males. The participants' ages ranged from 19 to 32 years (mean = 23.12; standard deviation = 4.38); most were over 20 years old. All participants were single and had not yet finished their undergraduate studies. Most worked part-time at the radio station and had never been trained in oral communication (Table 1).

**Table 1:** Sociodemographic and occupational characteristics of the sample in this study (n = 8)

Identification	n	%
**Sex**		
Females	5	62.5
Males	3	37.5
**Age**		
Under 20 years	1	12.5
Over 20 years	6	75
Over 30 years	1	12.5
**Marital status**		
Single	8	100
**Siblings**		
1 sibling	5	62.5
2 siblings	1	12.5
3 siblings	2	25
**Health problems in general**		
Yes	1	12.5
No	7	87.5
**Have you had or are you undergoing psychological treatment?**		
Yes	1	12.5
No	7	87.5
**Have you had or are you undergoing psychiatric treatment?**		
No	8	100
**Medication use**		
Yes	2	25
No	6	75
**Education level**		
Ongoing undergraduate studies	8	100
**Paid work**		
Yes, part time	5	62.5
Yes, full time	1	12.5
Yes, besides self-employed jobs	1	12.5
No	1	12.5
**Working days**		
2 days	1	12.5
3 days	4	50
4 days	1	12.5
6 days	1	12.5
None	1	12.5
**Monthly income**		
I do not know	3	37.5
I would rather not inform	3	37.5
Up to R$ 1,000.00	2	25
**Have you ever had any voice and/or speech training before?**		
Yes	1	12.5
No	7	87.5

### Procedures

The participants underwent the Program for Developing Oral Communication Expressiveness^(16)^, which comprised practical and theoretical training addressing various aspects of communication, such as speech, voice, interpretation, and oral expressiveness with vocal resources (Chart 1). Two research team members conducted the eight 2-hour intervention sessions in a climate-controlled classroom on the same campus as the radio station.

**Chart 1:** Description of the Program for Developing Oral Communication Expressiveness applied to study participants

**Session 1: Opening – Program bases (2 hours)**	
**Part I**	Presenting the training and researchers
	Providing an informed consent form
	Administering the self-assessment questionnaire
**Part II**	Recording individual voices
**Homework**	Perceiving their and other speakers’ oral communication
**Session 2: Breathing (2 hours)**	
**Part I (lasting 40 minutes)**	Participants rate their involvement in the homework assignment on a scale of 0 to 10
	Dialogical presentation on the basic mechanisms of voice production (notions of anatomy and physiology of vocal production) and basic principles of interpersonal communication
**Part II (lasting 20 minutes)**	Discussion on punctuation and breathing, in addition to performing a respiratory pause according to the logic of the text, with an example of a sentence with different meanings according to the punctuation used
	Cervical movements and shoulder rotation technique
	Large movements of the costodiaphragmatic region during sequences of deep inspirations and expirations
**Part III (lasting 20 minutes)**	Exercise in marking punctuation, such as commas and periods, in printed texts without graphic symbols in which the annotation must be made according to the logic of the text, noting the difference in the duration of the pauses in the case of commas and periods
	Reading texts aloud with brief individual feedback
**Part IV (lasting 40 minutes)**	Individual video recording of the informative text “Brazil, a country with a partially free press” read aloud for later analysis by participants
**Homework**	Carrying out exercises and reading texts aloud as proposed at the meeting
	Perception of their and other speakers’ oral communication
	Observation of breathing and its relationship with the content of what is said
**Session 3: Vocal warm-up (2 hours)**	
**Part I (lasting 60 minutes)**	Participants rate their involvement in the homework assignment on a scale of 0 to 10
	Dialogued presentation on vocal health and aspects related to the impressions transmitted by different vocal resources
**Part II (lasting 30 minutes)**	Reading aloud informational text
	Strategy to guide the participant in the overall understanding of the text through questions to identify the structure of the text and infer the author's intention
	Reading the same text aloud and comparing readings before and after understanding the text
	Cervical movements and shoulder rotation technique
	Large movements of the structures of the costodiaphragmatic region during sequences of deep inspirations and expirations
	Vibrant sound technique in sustained, modulated emissions and in musical scales
	Yawn-sigh technique
	Glottic firmness technique
	Nasal sound technique associated with masticatory technique
**Part III (lasting 30 minutes)**	Reading aloud the same informative text, analyzing situations
	First reading without discussion of the text
	Second reading, after discussing and understanding the text
	Third reading, after understanding the text and warming up the voice
	Comparison between readings and brief individual feedback
	Reading aloud another informative text with brief individual feedback, scoring parameters such as frequency, intensity, speech articulation, resonance, and their relationship with the content of the text
**Homework**	Performing exercises and reading aloud proposed at the meeting
	Perception of their and others' oral communication
	Observation of voice frequency and intensity, speech articulation and resonance, as well as the relationship with the content of what is said
**Session 4: Speech sound articulation (2 hours)**	
**Part I (lasting 50 minutes)**	Participants rate their involvement in homework and their oral communication during the week from 0 to 10
	Video presentation to show examples of vocal psychodynamics, a topic already discussed in the previous meeting in which the impressions transmitted by vocal resources were discussed
	Dialogical exhibition with audio and video examples showing people with different types of articulatory patterns
**Part II (lasting 30 minutes)**	Reading aloud informational text
	Questions to guide overall understanding of the text: identifying the structure of the text and inferring the author's intention
	Technique of cervical movements and shoulder rotation associated with the technique of vibrating sounds
	Vibrant sound technique in modulated emissions
	Nasal sound technique associated with masticatory technique
	Tongue rotation technique in the oral cavity associated with the nasal sound technique
	Masticatory technique
	Over articulation technique
**Part III (lasting 40 minutes)**	Reading aloud the same informative text and comparing readings before and after the exercises, with brief individual feedback
	Reading aloud an advertising text aimed at a young audience – therefore, to be read at a fast speech rate, maintaining articulatory precision
	Combination of all the skills worked on during this meeting: text comprehension strategies and exercises to ensure well-defined articulation
	Brief individual feedback
**Homework**	Performing exercises and reading texts aloud as proposed at the meeting
	Perception of their and other speakers’ oral communication
	Observing speech sound articulation and their relationship with the content of what is said
**Session 5: Frequency and intensity modulation (2 hours)**	
**Part I (lasting 50 minutes)**	Participants rate their involvement in homework and their oral communication during the week from 0 to 10
	Dialogical exhibition with audio and video examples showing people with different types of frequency and intensity modulation
	Audio example presentation to show the different vocal inflections according to the punctuation of the text
**Part II (lasting 30 minutes)**	Reading aloud text in which the same sentence has different meanings depending on the position of the comma
	Technique of cervical movements and shoulder rotation associated with the technique of vibrating sounds
	Nasal sound technique associated with masticatory technique
	Basal sound technique
	Blowing technique and high-pitched sound
	Vibrant sound technique in modulated emissions and musical scales
**Part III (lasting 40 minutes)**	Frequency and intensity modulation technique
	Reading special sentences to practice different inflections and with words previously marked to practice emphasis
	Reading poetry aloud
	Understand how each participant uses vocal resources according to their personal interpretation of the text and the message they wish to convey
	Brief individual feedback
**Homework**	Performing exercises and reading texts aloud as proposed at the meeting
	Perception of their and other speakers’ oral communication
	Observation of frequency and intensity modulation and its relationship with the content of what is said
**Session 6: Resonance (2 hours)**	
**Part I (lasting 20 minutes)**	Participants rate their involvement in homework and their oral communication during the week from 0 to 10
**Part II (lasting 30 minutes)**	Reading aloud advertising text
	Questions to guide overall understanding of the text: identifying the structure of the text and inferring the author's intention
	Reading informational text aloud before exercises
	Technique of cervical movements and shoulder rotation associated with the technique of vibrating sounds
	Fricative sounds technique: linked emission of voiced fricatives: “vzj vzj vzj”
	Yawn-sigh technique
	Nasal sound technique associated with masticatory technique
	Tongue rotation technique in the oral cavity associated with the nasal sound technique
**Part III (lasting 40 minutes)**	Reading aloud the same advertising text and comparing readings before and after exercises, with brief individual feedback
	Chanting voice technique associated with articulatory sequences and automatic speech
	Reading informative text aloud with brief individual feedback
	Combination of all the skills worked on during this meeting: text comprehension strategies and exercises to ensure balanced resonance and promote better vocal projection
**Part IV (lasting 30 minutes)**	Individual video recording of the informative text “Brazil, a country with a partially free press” read aloud (the same text used in Session 2) for analysis by participants
**Homework**	Performing exercises and reading texts aloud as proposed at the meeting
	Perception of their and other speakers’ oral communication
	Observation of resonance and its relationship with the content of what is said
**Session 7: Comparison of oral communication before and after training (2 hours)**	
**Part I (lasting 20 minutes)**	Participants rate their involvement in homework and their oral communication during the week from 0 to 10
**Part II (lasting 10 minutes)**	Dialogical exposition on verbal expressiveness in the text, drawing attention to the fact that voice and sound are always loaded with meaning, as well as reviewing all the vocal parameters addressed throughout vocal training, relating the impressions transmitted by the various voice resources
	Explaining how the next activity will occur – comparison between videos before and after training, individual comments, self-assessment, feedback from colleagues and the Speech-Language Pathologist
	Presenting each participant’s videos, organized in pairs, recorded in sessions 2 and 6, considered as pre- and post-training material, respectively
	Analysis of recordings
	Feedback provided immediately after watching each student's video
	Comments on the points that have improved and those that could still be improved
**Homework**	Performing the exercises proposed throughout the training, according to individual needs
	Perception of their and other speakers' oral communication
**Session 8: Concluding the program (2 hours)**	
**Part I (lasting 20 minutes)**	Participants rate their involvement in homework and their oral communication during the week from 0 to 10
**Part II (lasting 100 minutes)**	Summary of the training proposal, resuming exercises, and reinforcing the most important points
	Application of the self-assessment questionnaire
	Individual voice recording

In the first session, they presented the foundations of the program and administered assessment instruments. From the second to the seventh session, the structure had three distinct parts: Part I – dialogical presentation and auditory stimulation to develop communicative perception; Part II – implementing exercises and strategies for vocal preparation; and Part III – performing exercises and speech strategies, including short text reading, to improve oral communication skills.

The duration of each segment varied depending on its topic and activities. In the final session, they reviewed the topics addressed during the training and readministered the assessment instruments. This session structure enabled a comprehensive and progressive approach to developing the participants' oral communication expressiveness.

The announcers were assessed before and after the program using various evaluations, namely: a self-perception questionnaire, auditory-perceptual judgment conducted by Speech-Language Pathologists (SLP) specialized in voice, and acoustic analysis of their voices during standardized reading.

The self-perception of speech and voice was assessed with an instrument adapted from the Self-Assessment Questionnaire of Voice and Speech Skills in Various Communicative Contexts^(17)^. It retained the first 12 questions related to sociodemographic and occupational characteristics, along with two additional specific questions: “How do you perceive your diction (speech) when speaking in public?” and “How do you perceive the sound of your voice when speaking in public?” The response options for these two questions were, “The same as usual,” “Better than usual,” “Worse than usual,” and “Variable depending on the situation.”

The speech samples were recorded in the radio studio where the announcers worked, using the following standardized informative text (in Portuguese): “Jarbas Barbosa, the new president of ANVISA, advocates for changes in food packaging. The measure is necessary to facilitate the identification of products with high salt, sugar, or fat levels. This information is crucial to ensure all consumers have informed choices when shopping.” Participants were only introduced to this text in the first and final sessions. Female participants were asked about their menstrual cycle, and if the response was positive, the recording was rescheduled.

The recordings were made in an acoustically isolated studio, where noise levels were kept below 50 dB SPL, using a 44,000 Hz sampling rate and a 16-bit depth. The microphone used was an Audio-Technica model AT2020, equipped with a Shock Mount SH-100 anti-pop filter, connected by two Canon-Canon XLR cables, with an audio interface from Behringer, model UMC204 HD. The microphone was positioned at mouth level, maintaining an approximate distance of 5 centimeters. The samples were later edited using Audacity software, version 2.1.3, considering only the last sentence of the text for auditory-perceptual judgment.

The announcers' samples were randomized and labeled as “reading A” and “reading B,” with the researchers being the only ones aware of which moment each sample corresponded to. Three SLP judges, specialists in voice with at least 8 years of experience in the field, were invited to participate in the analysis of the readings. The judges evaluated the content independently and blindly, following the protocol guidelines used in its development study^(16)^.

The protocol for the auditory-perceptual judgment of voice and expressiveness had three distinct parts:

1. After listening to two files from the same announcer, labeled “Reading A” and “Reading B,” the SLP could mark “Similar” if she considered the two samples identical, or “Different” if she considered the readings different. If the samples were considered similar, the judge proceeded to Part II. If the samples were considered different, the judge was asked which one was better, Reading A or Reading B, and was requested to rate each one from 0 to 10. The protocol labels indicated criteria such as clear voice, sharp diction, message credibility, and engagement with the listener.
2. When the readings were considered similar, the judge classified the degree of vocal deviation in the samples into four categories: 0 for “no deviation,” 1 for “mild deviation,” 2 for “moderate deviation,” and 3 for “severe deviation.” If the samples were considered different, they followed the same process, but each reading was classified individually.
3. The judge analyzed the predominant vocal expressiveness features in the reading, such as pitch, intensity, speech rate, pauses, modulation, and emphasis, assigning a score from 0 to 2 for each item: 0 for “totally appropriate” to the text, 1 for “partially appropriate to the text,” and 2 for “inappropriate.” Similar readings were analyzed homogeneously, while different readings were analyzed separately.

The interjudge agreement was assessed with the multiple Kappa test – values less than 0 indicated insignificant agreement; 0 to 0.2, weak; 0.21 to 0.4, fair; 0.41 to 0.6, moderate; 0.61 to 0.8, strong; and 0.81 to 1, almost perfect or perfect agreement. Due to the low interjudge agreement, it was decided to consider only the judge with the highest internal coefficient – i.e., with a value of k = 1, indicating perfect agreement.

The acoustic measures were obtained from the speech samples using the Praat software (version 6.4.07), developed by Paul Boersma and David Weenink at the University of Amsterdam, Netherlands. Various measures were extracted, including the mean, median, standard deviation, minimum, and maximum of the fundamental frequency of speech (f0), as well as spectral slope (LTAS), Cepstral Peak Prominence (CPP), Cepstral Peak Prominence-Smoothed (CPPS), speech duration, articulation rate, speech rate, and intensity. The speech rate was calculated by annotating the words in the TextGrid layer, and dividing the total number of words by the total audio time. For articulation rate, pauses were identified and annotated, with the total pause time subtracted from the total audio time. The total number of words was then divided by the effective speaking time in minutes^(18)^.

### Statistical methods

Data were analyzed using descriptive statistical methods, employing both relative and absolute frequencies. Additionally, inferential statistical analyses were performed using RStudio software, version 2021.09.2. The Shapiro-Wilk test assessed the normality of the data distribution. The paired t-test compared continuous quantitative variables; however, when the data did not have a normal distribution, the Wilcoxon test for paired samples was applied. The Wilcoxon signed-rank test was employed for ordinal qualitative variables. The statistical significance level was set at p < 0.05.

## RESULTS

### Self-Perception of voice and speech

Regarding the question, “How do you perceive your diction (speech) when speaking in public?”, Table 2 shows that most participants reported that they considered their diction (speech) “variable depending on the situation” before the program. The percentage of participants who reported a “worsening of diction (speech)” decreased after the program, while half maintained the perception of “variable depending on the situation.”

**Table 2:** Comparison of self-perception variables of diction and voice sound when speaking in public before and after the intervention (n = 8)

Variable	Before		After		p-value*
	n	%	n	%	
**How do you perceive your diction (speech) when speaking in public?**					
Worse than usual	2	25	2	25	0.34
The same as usual	-	-	-	-	
Variable according to the situation	6	75	4	50	
Better than usual	-	-	2	25	
**How do you perceive the sound of your voice when speaking in public?**					
Worse than usual	-	-	2	25	0.17
The same as usual	1	12.5	1	12.5	
Variable according to the situation	7	-	3	37.5	
Better than usual	-	87.5	2	25	

* Wilcoxon rank test

**Caption:** n = number of subjects; % = percentage

As for the question, “How do you perceive the sound of your voice when speaking in public?”, most participants considered it “variable depending on the situation” before the program. As shown in Table 2, the percentage of participants who considered it “variable depending on the situation” clearly decreased after the program.

### Auditory-perceptual judgment of voice and expressiveness

Six (75%) of the eight pairs of readings showed differences, while two (25%) were considered identical. Two (33.33%) of the pairs identified as different (n = 6) were initially rated as superior, while four (66.67%) were judged as better after the training. The analysis of voice, speech, and interpretation influenced the selection in three (50%) of the divergent readings while interpretation was decisive in two (33.33%), and voice and speech played a crucial role in one (16.66%). Diction had the main change perceived in three readings (50%), followed by involvement (n = 2/33.33%) and credibility (n = 1/16.66%).

**Figure 1:**
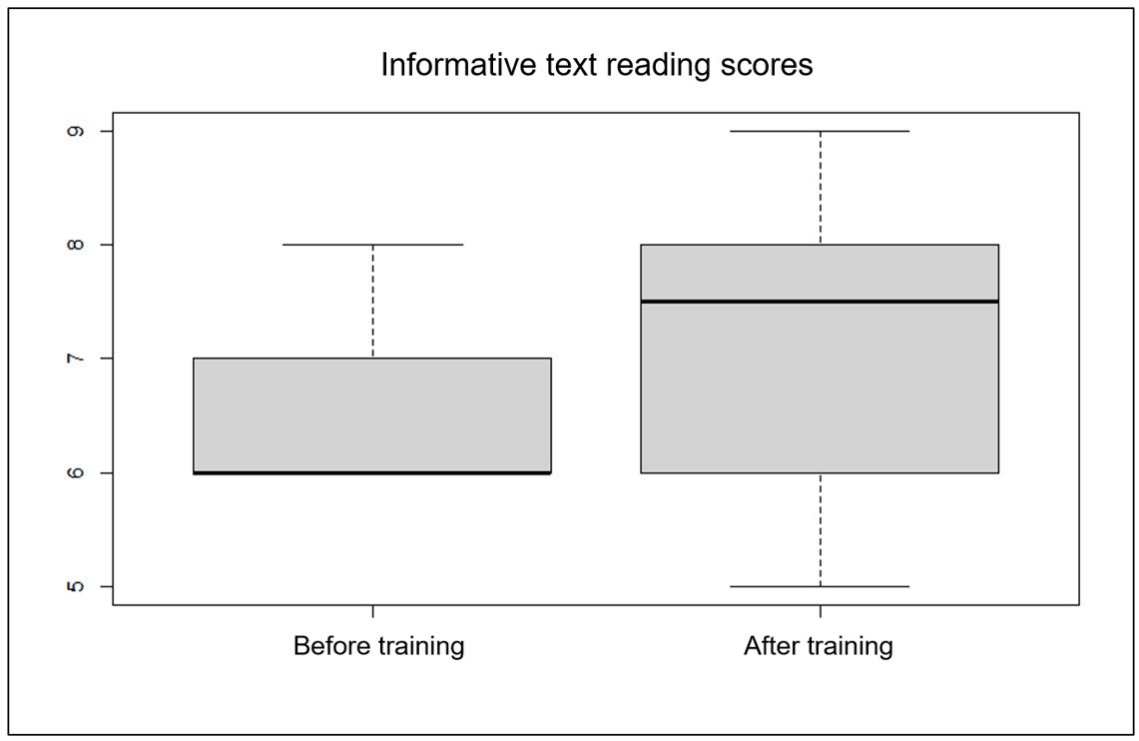
Boxplot analysis of variation in reading scores for informative text before and after training (n = 8)

The median and mean reading scores were higher after than before the program (p < 0.001), indicating a statistically significant difference between the groups before and after the program and a possible improvement in the quality of the reading following the intervention (Figure 1).

Moreover, most participants were classified as having mild vocal deviation before the program. This changed after the program, with half of the participants being classified as having no vocal deviation and the other half as having mild vocal deviation (Table 3).

**Table 3:** Comparison of vocal quality rating and vocal features before and after training (n = 8)

Variables	Before		After		p-value*
	n	%	n	%	
**Vocal deviation**					
Absent	3	37.5	4	50	0.77
Mild	5	62.5	4	50	
**Frequency**					
Inadequate	4	50	4	50	1
Partially adequate	1	12.5	1	12.5	
Adequate	3	37.5	3	37.5	
**Intensity**					
Inadequate	1	12.5	-	-	0.57
Partially adequate	5	62.5	5	62.5	
Adequate	2	25	3	37.5	
**Speech rate**					
Inadequate	2	25	3	37.5	0.85
Partially adequate	6	75	3	37.5	
Adequate	-	-	2	25	
**Pauses**					
Inadequate	7	87.5	5	62.5	0.34
Partially adequate	1	12.5	2	25	
Adequate	-	-	1	12.5	
**Modulation**					
Inadequate	1	12.5	3	37.5	0.76
Partially adequate	7	87.5	4	50	
Adequate	-	-	1	12.5	
**Emphasis**					
Inadequate	6	75	4	50	0.37
Partially adequate	2	25	3	37.5	
Adequate	-	-	1	12.5	

* Wilcoxon rank test

**Caption:** n = number of subjects; % = percentage

Similarly, other variables such as pitch, intensity, speech rate, pauses, modulation, and emphasis had noticeable changes in the proportions before and after the program, although none of the results reached statistical significance (Table 3).

### Acoustic analysis

Table 4 shows that the acoustic analysis measures before and after the training reveal several changes. The median and mean f0 decreased after the training, while the minimum (p < 0.01) and maximum (p < 0.02) f0 values increased. The articulation and speech rates also increased, while speech duration decreased. There was a significant increase in CPP after the training (p < 0.02), although with no significant difference in CPPS. Furthermore, speech intensity decreased significantly after the training (p < 0.01).

**Table 4:** Comparison of acoustic analysis parameters before and after training (n = 8)

Variable	Minimum	Quartile 1	Median	Mean	Quartile 3	Maximum	p-value
**Median f0**							
Before	145.39	145.39	211.39	195.08	219.85	248.39	0.92*
After	144.95	144.95	189.96	194.43	222.35	282.06	
**Mean f0**							
Before	151.57	151.57	212	201.54	222.37	268.80	0.51*
After	157.97	157.97	194.83	205.70	229.05	306.39	
**Standard deviation of f0**							
Before	21.79	21.79	33.66	33.66	38.29	59.97	0.07**
After	32.79	34.32	36.55	39.72	36.99	69.40	
**Minimum f0**							
Before	51.42	91.20	104.46	104.91	132.53	156.41	0.01**
After	107.96	107.96	117.13	129.47	143.64	186.37	
**Maximum f0**							
Before	217.26	217.26	286.98	289.58	319.71	421.40	0.02*
After	257.23	257.23	308.18	320.73	325.35	506.98	
**LTAS**							
Before	-13.79	-15.01	-15.97	-15.74	-15.97	-19.01	0.94**
After	-14.17	-14.88	-15.14	-15.33	-16.26	-16.26	
**Number of pauses**							
Before	3	3	4	4.50	5	9	0.13**
After	1	1	1	2.50	3.50	7	
**Articulation rate**							
Before	5.32	5.38	6.01	6.06	6.61	7.23	0.07**
After	5.43	6.16	6.53	6.39	6.84	6.84	
**Elocution rate**							
Before	2.32	2.34	2.62	2.64	2.88	3.15	0.07**
After	2.36	2.68	2.85	2.78	2.98	2.98	
**Duration**							
Before	5.39	5.9	6.55	6.51	7.24	7.32	0.07**
After	5.70	5.70	5.97	6.13	6.32	7.18	
**CPP**							
Before	11.26	13.81	13.82	16.42	17.75	24.72	0.02**
After	14.77	16.79	25.42	23.02	26.51	29.77	
**CPPS**							
Before	5.39	5.90	5.55	6.51	7.24	7.32	0.07**
After	5.70	5.70	5.97	6.13	6.32	7.18	
**Intensity**							
Before	66.62	66.62	67.53	67.80	68.28	69.98	**0.01** *
After	60.73	60.73	65.29	94.51	67.48	68.32	

Date in bold = statistical significance

* paired t-test;

** Wilcoxon test

**Caption:** f0 = fundamental frequency of speech; LTAS = Long-Term Average Spectrum; CPP = Cepstral Peak Prominence; CPPS = Cepstral Peak Prominence-Smoothed

## DISCUSSION

This pre/post intervention study offers valuable insights into the effects of the Program for Developing Oral Communication Expressiveness on radio announcers at a university radio station, who has been little studied by SLPs^(4,6,15)^. Despite the small sample size, often considered a limitation, it proves to be a crucial element in training oral communication competence in small groups^(19)^, allowing for individualized attention and more effective interaction among participants^(9,20)^. The analysis of the multidimensionality of oral communication assessment, including self-perception, auditory-perceptual judgment, and acoustic analysis, provides a comprehensive view of the training's impacts in this specific context.

The self-perception of voice and speech changed positively after the training, with a reduction in the number of participants reporting a worsening in diction and a considerable percentage indicating an improvement in the sound of their voice. This suggests that the announcers became more aware and confident regarding their own vocal expressiveness, which is crucial for more competent communication. The scientific literature reports that training to improve oral communication can enhance positive self-perception of voice and speech^(21,22)^. This improvement in self-perception may directly influence the quality of radio broadcasts, making the message more engaging for the audience^(3)^.

In the auditory-perceptual judgment of voice and expressiveness in text readings, most of the evaluated pairs showed differences, with half being considered better after the training. The analysis of voice, speech, and interpretation directly influenced the selection of the best readings, highlighting the importance of the ability to convey convincing and engaging messages^(8)^. This results in a natural and pleasant-sounding announcer style, demonstrating the ability to make vocal adjustments that suit the station's style^(3)^. These characteristics often distinguish them from the voices of nostalgic veterans^(3)^. In addition to vocal traits, the broadcast content and the announcer's personality are currently of even greater importance^(3)^. This underscores the program’s impact in developing technical aspects of oral communication and the announcers’ expressive and interpretative skills^(23)^.

The vocal resources of expressiveness that were positively modified may have influenced the improvement in reading, even if they did not reach statistical significance. When analyzing the variables before and after the intervention program, it is observed that some variables show slight improvement. For example, vocal pitch shows a similar distribution before and after the intervention, suggesting stability in vocal pitch. Additionally, speech intensity improved slightly after the program, indicating a positive effect on vocal modulation. Speech rate and use of pauses also improved, though not statistically significant, but with a positive trend after the intervention. Although these changes did not reach statistical significance, they may indicate a positive response to the training, reflecting a potential positive influence on reading quality^(6,24)^. These results highlight the importance of considering both statistical outcomes and vocal resource changes, which may help improve verbal and nonverbal communication^(19)^.

The acoustic analysis showed significant changes in f0, with a reduction in its median and mean after the intervention. This suggests that the announcers could improve their control over their vocal frequency, resulting in a more stable and consistent voice production^(25)^. This evolution in vocal quality, characterized by a smoother, more stable, and more controlled voice, is consistently associated with the effects of Semi-Occluded Vocal Tract Exercises (SOVTE), commonly applied during this program^(26)^. This effect may result from training the vocal tract muscles during SOVTE, leading to vocal economy^(27-29)^ – i.e., the practice of using the voice efficiently and sustainably, which is crucial for professionals who rely on their voice for work, such as singers, teachers, actors, and call center workers. Developing and maintaining vocal economy is essential to ensure the quality and durability of the voice over time, highlighting the importance of SOVTE as an effective ingredient in this context^(19,30)^. This suggests that the participants can apply the techniques learned during the intervention to improve their vocal production in a healthier manner^(31)^.

The minimum and maximum f0 values increased after the intervention, which suggests an expansion of the announcers' vocal range. In other words, the announcers have a greater capacity to vary their voice pitch, indicating an increase in their vocal range. This could result from strengthening the muscles and skills required to produce different tones and vocal inflections^(32)^. A specific speech and language training program was previously implemented for students in a radio announcer course. The acoustic analysis revealed an increase in the average f0 and an expansion in the frequency range after the training. These results indicated a richer reading modulation after the training^(33)^.

The articulation and speech rate also increased after the intervention. This could indicate a potential improvement in the fluency and clarity of the participants' speech. This improvement may be attributed to a combination of factors, including enhanced motor control, improved diction, and practice of specific elocution skills^(8)^. Time is a valuable resource in university radios, as the message must be conveyed through the speech signal within minutes or seconds. This is why journalists need strategies to achieve the exact timing in their spoken discourse. The rhythmic structure of speech is one of the main tools for journalists to succeed in this endeavor^(34)^. A study^(35)^ investigated the effects of implementing a workshop in radio classes on students' oral communication. The data showed that students' articulation, intonation, and oral expressiveness during the radio program broadcast improved due to training to speak with clarity and versatility. The results revealed that most students agreed that the radio workshop classes were important for enhancing their oral expressiveness. The students also agreed that they could express their ideas and improve their articulation and speech speed. Additionally, they stated that they could speak with more fluency and articulatory precision while practicing as radio announcers, newsreaders, and professional reporters, which increased their self-confidence. Typically, voices considered most suitable for radio broadcasting are those of professional and more experienced announcers^(36)^.

The significant increase in CPP measures stands out. This result suggests an improvement in vocal quality in terms of clarity and prominence of formant frequencies^(37)^. Vocal training can increase the CPP by improving vocal technique, reducing unwanted noise, and enhancing resonance and vocal projection. This leads to a clearer, more distinct, and higher-quality vocal production, reflected in a higher CPP^(38,39)^. However, it is important to note that no significant difference was found in the CPPS, indicating that the smoothness of the participants' voices remained stable throughout the training^(40)^.

There was a decrease in speech intensity after the training, and this can be interpreted in various ways due to the different factors involved in the vocal process. It is important to consider aspects such as vocal modulation and expressiveness when analyzing this decrease. For example, a reduction in speech intensity may indicate a change in the announcers’ vocal technique, leading to a more controlled and less strained vocal production. This can be interpreted positively, suggesting an improvement in vocal quality and a reduction in vocal fatigue^(12)^. However, it is also important to consider the modulation and expressiveness of the announcers' voices. A decrease in intensity without a corresponding reduction in vocal expressiveness may indicate a loss of dynamic range in speech^(41)^, which could affect communication quality. On the other hand, if the decrease in intensity is accompanied by appropriate modulation and consistent vocal expressiveness, it could indicate an adequate adjustment of vocal technique to different communication contexts^(8)^.

The program proved to be beneficial in improving the announcers’ communicative competence, which has significant implications for the training of professional radio announcers. These results highlight the importance of specific and multidimensional training programs to enhance communication competence in university radio announcers.

It is important to mention some limitations of this study so that the results are interpreted cautiously. One of them is the lack of a control group, which makes it difficult to directly attribute the observed changes to the intervention, as other uncontrolled factors may have influenced the results. Furthermore, a recent review of the scientific literature on SLP interventions with radio and TV professionals and students^(19)^ indicated that the most used designs were quasi-experimental and pre/post intervention studies. Given that interventions often take place in natural and real environments, such as radio studios, it is challenging to establish the necessary control conditions for a fully experimental design. Moreover, this design has a high risk of bias and the Hawthorne effect, in which participants alter some of their behaviors simply because they know they are being studied, regardless of the intervention.

The sample size also deserves attention, as a small sample may limit the ability to detect significant intervention effects^(19)^. The availability of students working at radio stations to participate in research may be restricted, especially in specific areas or studies that require time and commitment. Also, SLP interventions focused on communication competence are generally conducted with small groups^(42)^. Studies with large samples and multiple sessions may require the repeated recruitment of various groups or the simultaneous involvement of several SLP, which can be unfeasible, particularly when the intervention is carried out at the participants' workplace^(19)^.

Regarding the program duration and intensity, it is important to acknowledge that an intervention lasting only 2 hours over 8 weeks may be considered short or not intensive enough to produce significant changes in the announcers’ oral communication. However, this appears to be a time frame commonly used in SLP interventions with students working in radio^(19)^.

Additionally, the evaluation based solely on self-perception of speech and voice, auditory-perceptual judgment, and acoustic analysis may not encompass all relevant aspects of oral communication. The possibility of short-term effects should also be considered, as the results observed immediately after the intervention may not be maintained in the long term.

Finally, it is important to mention that there was no control over external variables, such as changes in the radio's programming or external events. Additionally, the differences between men and women were not analyzed separately, even though they have distinct anatomical and physiological configurations that may influence acoustic measures. It is essential to consider these limitations when interpreting and generalizing the results of this study.

## CONCLUSION

The effects of the Program for Developing Oral Communication Expressiveness for university radio announcers indicate improvements in the announcers’ communication skills, with a focus on self-perception of diction and voice quality. Half of the pairs improved the auditory-perceptual judgment of reading after the training, highlighting the program's potential to enhance voice quality, speech, and interpretation of informative text reading. The acoustic analysis found changes in speech f0, articulation rate, and speech rate, suggesting improved vocal quality and fluency. While no significant difference was found in voice smoothness, the decrease in speech intensity can be interpreted as an aspect of vocal modulation and expressiveness.
