# Supplementary material for: Effects of the Program for Developing Oral Communication Expressiveness on university radio announcers: a pre/post intervention study
Source: Codas. 2025 Feb 10;37(2):e20240122. doi: 10.1590/2317-1782/e20240122en (PMC11813182; doi:10.1590/2317-1782/e20240122en)
Supplement: Translation

# Translation: Efeitos do Programa de Desenvolvimento da Expressividade para Comunicação Oral em locutores de uma rádio universitária: um estudo antes e após intervenção

**DOI:** 10.1590/2317-1782/e20240122pt

**Authors:** Denis de Jesus Batista, Aline Santos da Conceição

**Affiliations:** 1 Programa de Pós-graduação em Modelos de Decisão e Saúde, Centro de Ciências Exatas e da Natureza, Universidade Federal da Paraíba – UFPB - João Pessoa (PB), Brasil.; 2 Dr Liang Voice Program, Faculty of Medicine and Health, The University of Sydney - Sidney, Australia.; 3 Centro Universitário Jorge Amado – Salvador (BA), Brasil.

**Keywords:** Fala, Rádio, Treinamento da Voz, Universidades, Voz

## Abstract

**Objetivo:**

analisar os efeitos do Programa de Desenvolvimento da Expressividade para Comunicação Oral em locutores de uma rádio universitária.

**Método:**

este é um estudo antes e após intervenção. Durante oito encontros de duas horas cada, foi implementado o Programa de Desenvolvimento da Expressividade para Comunicação Oral em oito locutores. Os participantes foram avaliados antes e depois do programa por instrumentos de autopercepção, julgamento perceptivo-auditivo e análise acústica de suas vozes durante leituras padronizadas. A análise estatística incluiu métodos descritivos e de inferência estatística com um nível de significância de p<0,05.

**Resultados:**

após o programa, houve uma melhoria na autopercepção dos locutores na dicção e na voz. No julgamento perceptivo-auditivo das leituras, a metade dos pares avaliados mostrou evolução, com destaque para a qualidade da voz, a fala e a interpretação. Na análise acústica, observou-se uma redução na frequência fundamental da fala, aumento na taxa de articulação e elocução, e diminuição da intensidade da fala.

**Conclusão:**

o programa mostrou considerável melhoria em aspectos da voz e da fala dos locutores, destacando a importância de programas de treinamento específicos para aprimorar as habilidades comunicativas em contextos radiofônicos.

## INTRODUÇÃO

As rádios universitárias desempenham um papel fundamental na comunicação acadêmica, cultural e social. Como espaços de experimentação e produção de conteúdo, essas emissoras proporcionam aos estudantes universitários a oportunidade de desenvolver competências comunicativas e artísticas, ao mesmo tempo em que servem como veículos de informação e entretenimento para a comunidade acadêmica e a sociedade em geral^(1,2)^.

A competência na comunicação oral é um aspecto crucial para o sucesso das transmissões radiofônicas, pois influencia diretamente a capacidade dos locutores de transmitir informações de forma clara, coerente e envolvente^(3)^. A definição de competência na comunicação pode variar conforme o sentido que se dê ao termo discursivo, mas, de modo geral, o termo “competência na comunicação” se refere à habilidade de uma pessoa em utilizar a língua de maneira eficaz e apropriada em diferentes contextos comunicativos. Isso envolve conhecer os princípios da comunicação verbal e não-verbal de forma adequada às diversas situações, como falar em público, participar de uma conversa informal, gravar um vídeo para as redes sociais, entre outras situações comunicativas^(4)^. Em outras palavras, uma pessoa com domínio da competência na comunicação é capaz de adaptar seu uso da língua de acordo com o contexto, o público e o propósito da interação, garantindo que sua mensagem seja clara, relevante e adequada à situação em que está inserida^(5)^. No contexto das rádios universitárias, onde os locutores muitas vezes são estudantes ainda em formação, a necessidade de um treinamento específico para o desenvolvimento dessa competência se torna ainda mais relevante^(4,6)^.

A literatura científica tem destacado a importância dos treinamentos para o aprimoramento da comunicação oral para diversos contextos profissionais^(7-9)^, incluindo a locução em rádios^(4,6,10-12)^ e no período de formação profissional, como a graduação^(13,14)^. Estudos mostram que intervenções direcionadas a este público podem resultar em efeitos positivos na qualidade vocal, na dicção, na modulação e em outros aspectos da comunicação oral, contribuindo para uma maior consciência e competência na transmissão de mensagens e na interação com o público^(4,6-10)^.

Diante desse contexto, torna-se evidente a necessidade de investigações científicas que explorem os efeitos de programas de desenvolvimento da comunicação oral em locutores de rádio universitária^(15)^. Um estudo de intervenção com essa população pode fornecer contribuições valiosas sobre os efeitos desses treinamentos, além de contribuir para o aprimoramento das práticas de formação e capacitação de futuros profissionais da voz. Assim, o presente estudo propõe-se a analisar os efeitos do Programa de Desenvolvimento da Expressividade para Comunicação Oral em locutores de uma rádio universitária, utilizando uma análise multidimensional que integra dados de autopercepção da voz e da fala, julgamento perceptivo-auditivo e análise acústica.

## MÉTODO

### Desenho do estudo

Este é um estudo antes e após intervenção.

### Aspectos éticos

O estudo teve início após a aprovação do Comitê de Ética e Pesquisa, conforme parecer nº 2.780.453 do protocolo CAAE 87624618.0.0000.0041, e após a obtenção do consentimento da rádio. Todos os participantes envolvidos no estudo concordaram com sua participação, manifestando sua aprovação por meio da assinatura do Termo de Consentimento Livre e Esclarecido (TCLE).

### Participantes

O convite para participação no estudo foi realizado durante uma reunião administrativa da rádio. Os critérios de inclusão consistiram em ser acadêmico e atuar no corpo de locução da rádio universitária. Por outro lado, foram aplicados critérios de exclusão, como apresentar déficit cognitivo ou dificuldades para compreender e realizar as avaliações e exercícios propostos, estar envolvido em treinamento comunicativo ou vocal durante o período do estudo, ter apresentado histórico recente de disfonia ou sintomas de disfonia; ter hábito de fumar; estar gestante; não participar de todas as etapas da pesquisa.

Inicialmente, a amostra foi composta por nove participantes. No entanto, houve uma desistência ao longo do processo, resultando em um total de oito locutores.

O estudo contou com a participação de oito locutores da rádio universitária, dos quais cinco eram do sexo feminino e três do sexo masculino. A faixa etária dos participantes variou de 19 a 32 anos (média=23,12; desvio-padrão=4,38), com a maioria tendo idade acima de 20 anos. Todos os participantes eram solteiros e possuíam escolaridade superior incompleta. A maioria trabalhava meio período na rádio e nunca tinha realizado treinamento focado na comunicação oral (Tabela 1).

**Tabela 1:** Características sociodemográficas e ocupacionais da amostra deste estudo (n=8)

Identificação	n	%
**Sexo**		
Feminino	5	62,5
Masculino	3	37,5
**Idade**		
Menor que 20 anos	1	12,5
Acima de 20 anos	6	75
Acima de 30 anos	1	12,5
**Estado Civil**		
Solteiro	8	100
**Tem irmãos**		
1 irmão	5	62,5
2 irmãos	1	12,5
3 irmãos	2	25
**Problemas de Saúde em geral**		
Sim	1	12,5
Não	7	87,5
**Já fez ou faz tratamento psicológico**		
Sim	1	12,5
Não	7	87,5
**Já fez ou faz tratamento psiquiátrico**		
Não	8	100
**Uso de medicamentos**		
Sim	2	25
Não	6	75
**Escolaridade**		
Superior incompleto	8	100
**Trabalho remunerado**		
Sim, meio período	5	62,5
Sim, tempo integral	1	12,5
Sim, além de trabalho autônomos	1	12,5
Não	1	12,5
**Dias de trabalho**		
2 dias	1	12,5
3 dias	4	50
4 dias	1	12,5
6 dias	1	12,5
Nenhum	1	12,5
**Renda mensal**		
Não sei	3	37,5
Prefiro não informar	3	37,5
Até R$ 1.000	2	25
**Já realizou algum treinamento para voz e/ou fala anteriormente**		
Sim	1	12,5
Não	7	87,5

### Procedimentos

Os participantes foram submetidos ao Programa de Desenvolvimento da Expressividade para Comunicação Oral^(16)^ composto por treinamentos práticos e teóricos abordando diversos aspectos da comunicação, como fala, voz, interpretação e expressividade oral com uso dos recursos vocais (Quadro 1). Durante a intervenção, foram realizados oito encontros, cada um com a duração de duas horas. Estes encontros foram conduzidos por dois membros da equipe de pesquisa em uma sala de aula climatizada, localizada no próprio campus onde a rádio estava situada.

**Quadro 1:** Descrição do Programa de Desenvolvimento da Expressividade para Comunicação Oral aplicado nos participantes deste estudo

**Encontro 1: Abertura – Bases do Programa (2 horas)**	
**Parte I**	Apresentação do treinamento e dos pesquisadores envolvidos
	Entrega do TCLE
	Aplicação do questionário de autoavaliação
**Parte II**	Gravação individual das vozes
**Tarefa de Casa**	Percepção de sua comunicação oral e de outros interlocutores
**Encontro 2: Respiração (2 horas)**	
**Parte I (40 minutos de duração)**	Os participantes dão nota de 0 a 10 para seu envolvimento na tarefa de casa
	Exposição dialogada sobre os mecanismos básicos de produção da voz (noções de anatomia e fisiologia da produção vocal) e princípios básicos da comunicação interpessoal
**Parte II (20 minutos de duração)**	Discussão sobre pontuação e respiração, além de realização de pausa respiratória de acordo com a lógica do texto, com exemplo de frase com diferentes significados de acordo com a pontuação empregada
	Técnica de movimentos cervicais e de rotação de ombros
	Movimentos amplos da região costodiafragmática durante sequências de inspirações e expirações profundas
**Parte III (20 minutos de duração)**	Exercício de marcação de pontuação, como vírgula e ponto, em textos impressos sem os sinais gráficos em que a anotação deve ser feita de acordo com a lógica do texto, notando a diferença de duração das pausas no caso da vírgula e do ponto final
	Leitura em voz alta dos textos com breve feedback individual
**Parte IV (40 minutos de duração)**	Gravação em vídeo, individual, de leitura em voz alta do texto informativo “Brasil, um país com imprensa parcialmente livre” para posterior análise dos participantes
**Tarefa de casa**	Realização de exercícios e leitura em voz alta propostos no encontro
	Percepção de sua comunicação oral e de outros interlocutores
	Observação da respiração e sua relação com o conteúdo do que é dito
**Encontro 3: Aquecimento vocal (2 horas)**	
**Parte I (60 minutos de duração)**	Participantes dão nota de 0 a 10 para seu envolvimento na tarefa de casa
	Exposição dialogada sobre saúde vocal e aspectos relacionados às impressões transmitidas por diferentes recursos vocais
**Parte II (30 minutos de duração)**	Leitura em voz alta de texto informativo
	Estratégia para orientar o participante na compreensão global do texto por meio de perguntas para identificar a estrutura do texto e inferir a intenção do autor
	Leitura em voz alta do mesmo texto e comparação entre leituras antes e após compreensão do texto
	Técnica de movimentos cervicais e de rotação de ombros
	Movimentos amplos das estruturas da região costodiafragmática durante sequências de inspirações e expirações profundas
	Técnica de sons vibrantes em emissões sustentadas, moduladas e em escalas musicais
	Técnica do bocejo-suspiro
	Técnica de firmeza glótica
	Técnica de sons nasais associada à técnica mastigatória
**Parte III (30 minutos de duração)**	Leitura em voz alta do mesmo texto informativo, análise das situações
	primeira leitura sem discussão do texto
	segunda leitura, após discussão e compreensão do texto
	terceira leitura, após compreensão do texto e após aquecimento vocal
	Comparação entre as leituras e breve feedback individual
	Leitura em voz alta de outro texto informativo com breve feedback individual, pontuando parâmetros como frequência, intensidade, articulação da fala e ressonância, além de sua relação com o conteúdo do texto
**Tarefa de casa**	Realização de exercícios e leitura em voz alta propostos no encontro
	Percepção de sua comunicação oral e de outros
	Observação da frequência e intensidade da voz, articulação da fala e ressonância, além da relação com o conteúdo do que é dito
**Encontro 4: Articulação dos sons da fala (2 horas)**	
**Parte I (50 minutos de duração)**	Participantes dão nota de 0 a 10 para seu envolvimento na tarefa de casa e para sua comunicação oral durante a semana
	Apresentação de vídeo para mostrar exemplos de psicodinâmica vocal, tema já comentado no encontro anterior em que foram discutidas as impressões transmitidas pelos recursos vocais
	Exposição dialogada com exemplos de áudios e vídeos que mostram pessoas com diferentes tipos de padrões articulatórios
**Parte II (30 minutos de duração)**	Leitura em voz alta de texto informativo
	Perguntas para direcionar a compreensão global do texto: identificação da estrutura do texto e inferência da intenção do autor
	Técnica de movimentos cervicais e de rotação de ombros associada à técnica de sons vibrantes
	Técnica de sons vibrantes em emissões moduladas
	Técnica de sons nasais associada à técnica mastigatória
	Técnica de rotação de língua no vestíbulo bucal associada à técnica de sons nasais
	Técnica mastigatória
	Técnica de sobrearticulação
**Parte III (40 minutos de duração)**	Leitura em voz alta do mesmo texto informativo e comparação entre leituras antes e após os exercícios, com breve feedback individual
	Leitura em voz alta de um texto publicitário que é dirigido a um público jovem, portanto, a ser lido com velocidade rápida de fala, mantendo precisão articulatória; Reunião de todas as habilidades trabalhadas durante este encontro: estratégias de compreensão do texto e exercícios para garantir uma articulação bem definida
	Breve feedback individual
**Tarefa de casa**	Realização de exercícios e leitura em voz alta propostos no encontro
	Percepção de sua comunicação oral e de outros
	Observação da articulação dos sons da fala e sua relação com o conteúdo do que é dito
**Encontro 5: Modulação de frequência e intensidade (2 horas)**	
**Parte I (50 minutos de duração)**	Participantes dão nota de 0 a 10 para seu envolvimento na tarefa de casa e para sua comunicação oral durante a semana
	Exposição dialogada com exemplos de áudios e vídeos que mostram pessoas com diferentes tipos de modulação de frequência e intensidade
	Apresentação de exemplo de áudio para mostrar as diferentes inflexões vocais de acordo com a pontuação do texto
**Parte II (30 minutos de duração)**	Leitura em voz alta de texto em que a mesma frase tem sentidos distintos de acordo com a posição do sinal gráfico de vírgula
	Técnica de movimentos cervicais e de rotação de ombros associada à técnica de sons vibrantes
	Técnica de sons nasais associada à técnica mastigatória
	Técnica de som basal
	Técnica de sopro e som agudo
	Técnica de sons vibrantes em emissões moduladas e em escalas musicais
**Parte III (40 minutos de duração)**	Técnica de modulação de frequência e intensidade; Leitura de frases especiais para treino de diferentes inflexões e com palavras previamente marcadas para exercício de ênfases
	Leitura em voz alta de poesia; Perceber como cada participante emprega os recursos vocais de acordo com sua interpretação pessoal do texto e a mensagem que deseja transmitir; Breve feedback individual.
**Tarefa de casa**	Realização de exercícios e leitura em voz alta propostos no encontro
	Percepção de sua comunicação oral e de outros interlocutores
	Observação da modulação de frequência e intensidade e sua relação com o conteúdo do que é dito
**Encontro 6: Ressonância (2 horas)**	
**Parte I (20 minutos de duração)**	Participantes dão nota de 0 a 10 para seu envolvimento na tarefa de casa e para sua comunicação oral durante a semana
**Parte II (30 minutos de duração)**	Leitura em voz alta de texto publicitário
	Perguntas para direcionar a compreensão global do texto: identificação da estrutura do texto e inferência da intenção do autor
	Leitura em voz alta de texto informativo antes dos exercícios
	Técnica de movimentos cervicais e de rotação de ombros associada à técnica de sons vibrantes
	Técnica de sons fricativos: emissão de fricativos sonoros de forma concatenada “vzj vzj vzj”
	Técnica do bocejo-suspiro
	Técnica de sons nasais associada à técnica mastigatória
	Técnica de rotação de língua no vestíbulo bucal associada à técnica de sons nasais
**Parte III (40 minutos de duração)**	Leitura em voz alta do mesmo texto publicitário e comparação entre leituras antes e após exercícios, com breve feedback individual
	Técnica de voz salmodiada associada a sequências articulatórias e à fala automática
	Leitura em voz alta de texto informativo com breve feedback individual; Reunião de todas as habilidades trabalhadas durante este encontro: estratégias de compreensão do texto e exercícios para garantir uma ressonância equilibrada e promover melhor projeção vocal
**Parte IV (30 minutos de duração)**	Gravação em vídeo, individual, de leitura em voz alta do texto informativo “Brasil, um país com imprensa parcialmente livre”, mesmo texto utilizado no Encontro 2, para análise dos participantes
**Tarefa de casa**	Realização de exercícios e leitura em voz alta propostos no encontro
	Percepção de sua comunicação oral e de outros interlocutores
	Observação da ressonância e sua relação com o conteúdo do que é dito
**Encontro 7: Comparação da comunicação oral antes e após treinamento (2 horas)**	
**Parte I (20 minutos de duração)**	Participantes dão nota de 0 a 10 para seu envolvimento na tarefa de casa e para sua comunicação oral durante a semana
**Parte II (10 minutos de duração)**	Exposição dialogada sobre a expressividade verbal no texto, chamando a atenção para a questão de que a voz e o som estão sempre carregados de sentido, bem como revisão de todos os parâmetros vocais trabalhados ao longo do treinamento vocal, relacionando as impressões transmitidas pelos diversos recursos de voz
	Explicação de como acontecerá a dinâmica seguinte – comparação entre vídeos antes e após treinamento, comentários individuais, autoavaliação, feedback dos colegas e da fonoaudióloga
	Apresentação dos vídeos de cada participante, organizados aos pares, gravados nos encontros 2 e 6, e considerados como material antes e após treinamento, respectivamente
	Análise das gravações
	Feedbacks feitos imediatamente após observação do vídeo de cada aluno
	Comentários sobre os pontos que melhoraram e aqueles que ainda poderiam ser aprimorados
**Tarefa de casa**	Realização dos exercícios propostos ao longo do treinamento, de acordo com a necessidade individual
	Percepção de sua comunicação oral e de outros
**Encontro 8: Finalização do programa (2 horas)**	
**Parte I (20 minutos de duração)**	Participantes dão nota de 0 a 10 para seu envolvimento na tarefa de casa e para sua comunicação oral durante a semana
**Parte II (100 minutos de duração)**	Resumo da proposta do treinamento, retomada dos exercícios e reforço dos pontos mais importantes
	Aplicação do questionário de autoavaliação
	Gravação individual das vozes

No primeiro encontro, foram apresentadas as bases do programa, além da aplicação dos instrumentos de avaliação. A partir do segundo até o sétimo encontro, o funcionamento foi estruturado em três partes distintas: Parte I - exposição dialogada e estímulo auditivo para o desenvolvimento da percepção comunicativa; Parte II - aplicação de exercícios e estratégias para a preparação vocal; e Parte III - realização de exercícios e aplicação de estratégias de fala, incluindo leitura de textos breves e curtos, visando o aprimoramento da competência na comunicação oral.

A duração de cada bloco variava conforme o tema abordado e as atividades propostas. No último encontro, ocorria uma revisão dos tópicos abordados durante o treinamento, seguida pela última aplicação dos instrumentos de avaliação. Essa estruturação dos encontros permitiu uma abordagem abrangente e progressiva no desenvolvimento da expressividade na comunicação oral dos participantes.

Antes e depois do programa, os locutores foram avaliados por meio de diversas avaliações, as quais incluíam: um questionário de autopercepção, julgamento perceptivo-auditiva realizada por fonoaudiólogas especializadas em voz e análise acústica de suas vozes durante leituras padronizadas.

Para a autopercepção do som da fala e da voz, foi utilizado um instrumento adaptado do questionário de Autoavaliação das Habilidades de Voz e Fala em Diversos Contextos Comunicativos^(17)^, mantendo as 12 primeiras questões relacionadas às características sociodemográficas e ocupacionais, além de outras duas questões específicas: “Ao falar em público, como é a autopercepção da sua dicção (fala)?” e “Ao falar em público, como é a autopercepção do som da sua voz?”. As opções de resposta para essas duas questões foram: “Igual a de sempre”, “Melhor do que a de sempre”, “Pior do que a de sempre” e “Variável de acordo com a situação”.

As amostras de fala foram gravadas no estúdio da rádio onde os locutores trabalhavam, utilizando um texto informativo padronizado: *“Jarbas Barbosa, novo presidente da Anvisa, defende mudanças na embalagem dos alimentos. A medida é necessária para facilitar a identificação de produtos com alto teor de sal, açúcar ou gordura. Essas informações são fundamentais para garantir a escolha consciente de todos os consumidores na hora de comprar”.* Os participantes tiveram contato com este texto apenas no primeiro e no último encontro. As participantes do sexo feminino foram questionadas sobre o ciclo menstrual. Em caso de confirmação positiva, a gravação era reagendada.

As gravações foram realizadas em um estúdio com isolamento acústico, onde o ruído foi mantido abaixo de 50 dB NPS. Foi utilizada uma taxa de amostragem de 44000 Hz e profundidade de 16 bits. O microfone utilizado foi da marca *Audio-technica*, modelo AT2020, equipado com filtro *anti-puff Shock Mount SH-100*, conectado por dois cabos *XLR* da marca *Canon-Canon*. Além disso, foi utilizado uma placa de interface de áudio da marca *Behringer*, modelo UMC204 HD. O microfone foi posicionado à altura da boca, mantendo uma distância aproximada de 5 centímetros. As amostras foram posteriormente editadas utilizando o *software Audacity*, versão 2.1.3, considerando apenas a última frase do texto para julgamento perceptivo-auditiva.

As amostras dos locutores foram aleatorizadas e designadas como “leitura A” e “leitura B”, com os pesquisadores sendo os únicos cientes de qual momento essas amostras pertenciam. Três juízas fonoaudiólogas, especialistas em voz, e com experiência mínima de oito anos na área, foram convidadas a participar da análise das leituras. As juízas avaliaram o conteúdo de forma independente e cega, seguindo as orientações do protocolo utilizado no estudo que o desenvolveu^(16)^.

O protocolo para o julgamento perceptivo-auditivo da voz e da expressividade consistia de três partes distintas:

1. Após ouvir dois arquivos do mesmo locutor, denominados “Leitura A” e “Leitura B”, respectivamente, a fonoaudióloga tinha a opção de marcar “Semelhantes” se considerasse as duas amostras iguais, ou “Diferentes” se considerasse as leituras distintas. Em caso de considerá-las semelhantes, a juíza seguia para a parte II. Se as amostras fossem consideradas diferentes, a juíza era questionada sobre qual era a melhor, Leitura A ou Leitura B, e solicitava-se uma nota de 0 a 10 para cada uma delas. As legendas no protocolo indicavam critérios como voz limpa, dicção nítida, credibilidade da mensagem e envolvimento com o ouvinte.
2. Quando as leituras eram consideradas semelhantes, a juíza classificava o grau de desvio vocal das amostras em quatro categorias: 0 para “ausência de desvio”, 1 para “desvio leve”, 2 para “desvio moderado” e 3 para “desvio intenso”. Se as amostras fossem consideradas diferentes, o mesmo processo ocorria, mas cada leitura era classificada individualmente.
3. A juíza analisava os recursos vocais da expressividade predominantes na leitura, como frequência, intensidade, velocidade de fala, pausas, modulação e ênfases, atribuindo uma pontuação de 0 a 2 para cada item: 0 para “totalmente adequado” ao texto; 1 para “parcialmente adequado ao texto” e 2 para “inadequado”. As leituras semelhantes eram analisadas de forma homogênea, enquanto as diferentes eram analisadas separadamente.

Para avaliar a concordância entre as juízas, foi utilizado o teste múltiplo de *Kappa*, com valores menores que zero indicando uma concordância insignificante, de 0 a 0,2 (fraco), de 0,21 a 0,4 (razoável), de 0,41 a 0,6 (moderado), de 0,61 a 0,8 (forte), e de 0,81 a 1 (quase perfeitos ou perfeitos). Devido ao baixo valor de concordância entre as juízas, optou-se por considerar apenas o julgamento da juíza com o maior coeficiente interno, neste caso, com o valor de *k*=1, indicando uma concordância perfeita.

As medidas acústicas foram obtidas das amostras de fala utilizando o *software Praat* (versão 6.4.07), desenvolvido por Paul Boersma e David Weenink, da *University of Amsterdam*, Holanda. Diversas medidas foram extraídas, incluindo: média, mediana, desvio-padrão, mínimo e máximo da frequência fundamental da fala (f0), além do declínio espectral (Ltas), *Cepstral Peak Prominence* (CPP) e *Cepstral Peak Prominence-Smoothed* (CPPS), duração da elocução, taxa de articulação e elocução, e intensidade. A taxa de elocução foi calculada pela anotação das palavras na camada *TextGrid*, dividindo-se o número total de palavras pelo tempo total do áudio. Para a taxa de articulação, foram identificadas e anotadas as pausas, subtraindo-se o tempo total das pausas do tempo total do áudio. O número total de palavras foi então dividido pelo tempo efetivo de fala em minutos^(18)^.

### Métodos estatísticos

A análise dos dados foi conduzida por meio de métodos estatísticos descritivos, utilizando frequências relativas e absolutas. Além disso, foram realizadas análises estatísticas inferenciais utilizando o software *RStudio*, versão 2021.09.2. Para avaliar a normalidade da distribuição dos dados, aplicou-se o teste de *Shapiro-Wilk*. Para comparar as variáveis quantitativas contínuas, utilizou-se o teste t pareado, no entanto, quando os dados não apresentaram distribuição normal, foi utilizado o teste de *Wilcoxon* para amostras pareadas. Já para as variáveis qualitativas ordinais, empregou-se o teste de postos com sinais de *Wilcoxon*. O nível de significância estatística adotado foi de p<0,05.

## RESULTADOS

### Autopercepção da voz e da fala

Para a questão “Ao falar em público, como é a autopercepção da sua dicção (fala)?”, observa-se na Tabela 2 que antes do programa, a maioria dos participantes relatam que consideram sua dicção (fala) “variável de acordo com a situação”. Após o programa, houve uma redução na porcentagem de participantes que relatam “piora na dicção (fala)”, enquanto a metade manteve a percepção “variável de acordo com a situação”.

**Tabela 2:** Comparação das variáveis de autopercepção da dicção e do som da voz ao falar em público antes e após a intervenção (n=8)

Variável	Antes		Depois		p-valor*
	n	%	n	%	
**Ao falar em público, como é a autopercepção da sua dicção (fala)**					
Pior do que a de sempre	2	25	2	25	0,34
Igual a de sempre	-	-	-	-	
Variável de acordo com a situação	6	75	4	50	
Melhor do que a de sempre	-	-	2	25	
**Ao falar em público, como é a autopercepção do som da sua voz**					
Pior do que a de sempre	-	-	2	25	0,17
Igual a de sempre	1	12,5	1	12,5	
Variável de acordo com a situação	7	-	3	37,5	
Melhor do que a de sempre	-	87,5	2	25	

* Teste de postos de Wilcoxon

**Legenda:** n=número de sujeitos; %=porcentagem

Quanto à questão “Ao falar em público, como é a autopercepção do som da sua voz?”, antes do programa, a maioria considerava “variável de acordo com a situação”. Após o programa, na Tabela 2, torna-se evidente uma redução na porcentagem de participantes que consideram “variável de acordo com a situação”.

### Julgamento perceptivo-auditivo da voz e da expressividade

Entre os oito pares de leituras avaliados, seis (75%) apresentam diferenças, enquanto dois (25%) foram considerados idênticos. Dos pares identificados como distintos (n=6), dois (33,33%) foram avaliados como superiores inicialmente, enquanto quatro (66,67%) foram julgados como melhores após o treinamento. A análise das variáveis voz, fala e interpretação influencia na seleção em três (50%) das leituras divergentes, enquanto em duas (33,33%) a interpretação foi determinante, e em uma (16,66%) a voz e a fala desempenham papel crucial. A principal mudança percebida em três leituras foi na dicção (50%), seguida pelo envolvimento (n=2/33,33%) e pela credibilidade (n=1/16,66%).

Para a variável “Nota das leituras”, observa-se que a mediana e a média das notas atribuídas às leituras foram maiores após o programa em comparação com antes (p<0,001), indicando uma diferença estatisticamente significativa entre os grupos antes e depois do programa e uma possível melhora na qualidade da leitura após a intervenção (Figura 1).

**Figura 1:** Análise por *boxplot* da variação das notas das leituras do texto informativo antes e após o treinamento (n=8)

Para a variável “Desvio Vocal”, observa-se que antes do programa, a maioria dos participantes foram classificados como tendo desvio vocal leve. Após o programa, houve uma mudança, com a metade dos participantes sendo classificados com ausência de desvio vocal e metade com leve desvio vocal (Tabela 3).

**Tabela 3:** Comparação da classificação da qualidade vocal e dos recursos vocais antes e após o treinamento (n=8)

Variáveis	Antes		Depois		p-valor*
	n	%	n	%	
Desvio Vocal					
Ausente	3	37,5	4	50	0,77
Leve	5	62,5	4	50	
Frequência					
Inadequado	4	50	4	50	1
Parcialmente adequado	1	12,5	1	12,5	
Adequado	3	37,5	3	37,5	
Intensidade					
Inadequado	1	12,5	-	-	0,57
Parcialmente adequado	5	62,5	5	62,5	
Adequado	2	25	3	37,5	
Velocidade de fala					
Inadequado	2	25	3	37,5	0,85
Parcialmente adequado	6	75	3	37,5	
Adequado	-	-	2	25	
Pausas					
Inadequado	7	87,5	5	62,5	0,34
Parcialmente adequado	1	12,5	2	25	
Adequado	-	-	1	12,5	
Modulação					
Inadequado	1	12,5	3	37,5	0,76
Parcialmente adequado	7	87,5	4	50	
Adequado	-	-	1	12,5	
Ênfases					
Inadequado	6	75	4	50	0,37
Parcialmente adequado	2	25	3	37,5	
Adequado	-	-	1	12,5	

* Teste de postos de Wilcoxon

**Legenda:** n = número de sujeitos; % = porcentagem

Da mesma forma, para as outras variáveis como frequência, intensidade, velocidade de fala, pausas, modulação e ênfases, percebe-se algumas mudanças nas proporções antes e depois do programa, mas nenhum resultado atingiu relevância estatística (Tabela 3).

### Análise acústica

Na Tabela 4, percebe-se que as medidas da análise acústica antes e após o treinamento revelam diversas mudanças. Após o treinamento, observa-se uma redução na mediana e média da frequência fundamental (f0), enquanto o mínimo (p<0,01) e o máximo da f0 (p<0,02) aumentam. As taxas de articulação e elocução também aumentam, enquanto a duração da fala diminui. Houve um aumento significativo no CPP após o treinamento (p<0,02), no entanto, não houve diferença significativa no CPPS. Destaca-se ainda que a intensidade da fala diminui significativamente após o treinamento (p<0,01).

**Tabela 4:** Comparação dos parâmetros da análise acústica antes e após o treinamento (n=8)

Variável	Mínimo	1º quartil	Mediana	Média	3º quartil	Máximo	p-valor
**Mediana da f0**							
antes	145,39	145,39	211,39	195,08	219,85	248,39	0,92*
após	144,95	144,95	189,96	194,43	222,35	282,06	
**Média da f0**							
antes	151,57	151,57	212	201,54	222,37	268,80	0,51*
após	157,97	157,97	194,83	205,70	229,05	306,39	
**Desvio padrão da f0**							
antes	21,79	21,79	33,66	33,66	38,29	59,97	0,07**
após	32,79	34,32	36,55	39,72	36,99	69,40	
**Mínimo da f0**							
antes	51,42	91,20	104,46	104,91	132,53	156,41	0,01**
após	107,96	107,96	117,13	129,47	143,64	186,37	
**Máximo da f0**							
antes	217,26	217,26	286,98	289,58	319,71	421,40	0,02*
após	257,23	257,23	308,18	320,73	325,35	506,98	
**Ltas**							
antes	-13,79	-15,01	-15,97	-15,74	-15,97	-19,01	0,94**
após	-14,17	-14,88	-15,14	-15,33	-16,26	-16,26	
**Número de pausas**							
antes	3	3	4	4,50	5	9	0,13**
após	1	1	1	2,50	3,50	7	
**Taxa de articulação**							
antes	5,32	5,38	6,01	6,06	6,61	7,23	0,07**
após	5,43	6,16	6,53	6,39	6,84	6,84	
**Taxa de elocução**							
antes	2,32	2,34	2,62	2,64	2,88	3,15	0,07**
após	2,36	2,68	2,85	2,78	2,98	2,98	
**Duração**							
antes	5,39	5,9	6,55	6,51	7,24	7,32	0,07**
após	5,70	5,70	5,97	6,13	6,32	7,18	
**CPP**							
antes	11,26	13,81	13,82	16,42	17,75	24,72	**0,02** **
após	14,77	16,79	25,42	23,02	26,51	29,77	
**CPPS**							
antes	5,39	5,90	5,55	6,51	7,24	7,32	0,07**
após	5,70	5,70	5,97	6,13	6,32	7,18	
**Intensidade**							
antes	66,62	66,62	67,53	67,80	68,28	69,98	**0,01** *
após	60,73	60,73	65,29	94,51	67,48	68,32	

Dados em negrito = significância estatística

* Teste t pareado;

** Teste de Wilcoxon

**Legenda:** f0 = frequência fundamental da fala; Ltas = Long-Term Average Spectrum; CPP = *Cepstral Peak Prominence*; CPPS = *Cepstral Peak Prominence-Smoothed*

## DISCUSSÃO

Este estudo, realizado antes e depois da intervenção, oferece valiosas contribuições sobre os efeitos do Programa de Desenvolvimento da Expressividade para Comunicação Oral em locutores de uma rádio universitária, uma população pouco estudada pela Fonoaudiologia^(4,6,15)^. Apesar do tamanho reduzido da amostra, frequentemente visto como uma limitação, ele se revela um elemento crucial para conduzir treinamentos direcionados à competência da comunicação oral em grupos pequenos^(19)^, permitindo uma atenção individualizada e uma interação mais eficaz entre os participantes^(9,20)^. A análise da multidimensionalidade da avaliação da comunicação oral, incluindo autopercepção, julgamento perceptivo-auditivo e análise acústica, proporciona uma visão abrangente dos impactos do treinamento nesse contexto específico.

Em relação à autopercepção da voz e da fala, observa uma mudança positiva após o treinamento, com uma redução no número de participantes relatando piora na dicção e uma porcentagem considerável indicando melhora no som da voz. Isso sugere que os locutores se tornaram mais conscientes e confiantes em relação à sua própria expressividade vocal, o que é fundamental para uma comunicação mais competente. A literatura científica relata que os treinamentos para o aperfeiçoamento da comunicação oral podem aumentar a autopercepção positiva sobre a voz e fala^(21,22)^. Essa melhoria na autopercepção pode influenciar diretamente a qualidade das transmissões radiofônicas, tornando a mensagem mais envolvente para o público^(3)^.

No julgamento perceptivo-auditivo da voz e da expressividade nas leituras de texto, a maioria dos pares avaliados apresentou diferenças, sendo que a metade foi considerada melhor após o treinamento. A análise das variáveis voz, fala e interpretação demonstra influenciar diretamente na seleção das melhores leituras, enfatizando a importância da habilidade em transmitir a mensagem de forma convincente e envolvente^(8)^. Ao que resultará num padrão de locução atual apresentando uma sonoridade natural e agradável, demonstrando habilidade para realizar ajustes vocais que se adequam ao estilo da estação^(3)^. Essas características frequentemente os distinguem das vozes de saudosistas da velha guarda^(3)^. Atualmente, além das características vocais, o conteúdo transmitido e a personalidade do locutor assumem uma importância ainda maior^(3)^. Isso ressalta o impacto do programa em desenvolver não apenas aspectos técnicos da comunicação oral, mas também habilidades expressivas e interpretativas dos locutores^(23)^.

É importante notar que os recursos vocais da expressividade que foram modificados positivamente podem ter influenciado a melhoria da leitura, mesmo que não tenham alcançado significância estatística. Ao analisar as variáveis antes e depois do programa de intervenção, observa-se que algumas variáveis mostram uma discreta melhora. Por exemplo, a frequência vocal mostra uma distribuição similar antes e depois da intervenção, sugerindo uma estabilidade na frequência vocal. Além disso, a intensidade da fala apresenta uma leve melhoria após o programa, indicando um efeito positivo na modulação vocal. Da mesma forma, a velocidade de fala e o uso de pausas também mostram melhorias não significativas, mas com uma melhora positiva após a intervenção. Embora essas mudanças não tenham atingido significância estatística, elas podem indicar uma resposta positiva ao treinamento, refletindo uma possível influência positiva na qualidade da leitura^(6,24)^. Esses resultados destacam a importância de considerar não apenas os resultados estatísticos, mas também as mudanças observadas nos recursos vocais, que podem contribuir para aprimorar a comunicação verbal e não-verbal^(19)^.

Por meio de análise acústica, observa-se mudanças significativas na frequência fundamental da fala (f0), com uma redução na mediana e média da f0 após a intervenção. Essa diminuição na mediana e média da f0 sugere que os locutores foram capazes de melhorar o controle sobre sua frequência vocal, resultando em uma produção de voz mais estável e consistente^(25)^. Essa evolução na qualidade vocal, caracterizada por uma voz mais estável, suave e controlada, é constantemente associada aos efeitos dos exercícios de trato vocal semi-ocluído (ETVSO) comumente aplicados durante este programa^(26)^. Este efeito pode ser resultado do treinamento dos músculos do trato vocal durante os ETVSO, levando a uma economia vocal^(27-29)^. A economia vocal refere-se à prática de usar a voz de forma eficiente e sustentável, o que é crucial para profissionais que dependem da voz para seu trabalho, como cantores, professores, atores e profissionais de *callcenter*. Desenvolver e manter uma economia vocal é essencial para garantir a qualidade e durabilidade da voz ao longo do tempo, destacando a importância dos ETVSO como um ingrediente eficaz nesse contexto^(19,30)^. Isso sugere que os participantes são capazes de aplicar as técnicas aprendidas durante a intervenção para melhorar sua produção vocal de forma mais saudável^(31)^.

Os valores mínimo e máximo da f0 aumentaram após a intervenção. Estes dados sugerem uma ampliação da extensão vocal dos locutores. Em outras palavras, os locutores têm uma capacidade maior de variar a altura da voz, o que sugere uma expansão da sua extensão vocal. Isso pode ser resultado do fortalecimento dos músculos e das habilidades necessárias para produzir diferentes tons e inflexões vocais^(32)^. Previamente, um programa específico de treinamento em fala e linguagem foi implementado em alunos de um curso de locução radiofônica. A análise acústica revelou um aumento na frequência fundamental média e uma expansão na faixa de frequência após o treinamento. Esses resultados indicaram uma modulação mais rica nas leituras após o treinamento^(33)^.

As taxas de articulação e elocução também aumentaram após a intervenção. Isso pode indicar uma possível melhora na fluência e clareza da fala dos participantes. Essa melhora pode ser atribuída a uma combinação de fatores, incluindo controle motor aprimorado, aprimoramento da dicção e prática de habilidades específicas de elocução^(8)^. O tempo é um recurso valioso nas rádios universitárias, pois a mensagem deve ser transmitida pelo sinal de fala em poucos minutos ou segundos. É por esse motivo que os jornalistas precisam de estratégias para atingirem o *timing* exato do seu discurso falado. A estrutura rítmica do discurso é uma das principais ferramentas para os jornalistas terem sucesso nesta empreitada^(34)^. Um estudo^(35)^ investigou os efeitos da implementação de um *workshop* nas aulas de rádio na comunicação oral dos alunos. Os dados demonstram que a articulação, entonação e expressividade oral dos alunos na transmissão do programa de rádio melhoraram devido ao treinamento para falar com clareza e versatilidade. Os resultados revelaram que a maioria dos alunos concorda que as aulas do *workshop* sobre rádio são importantes para aprimorar sua capacidade de expressividade oral. Os alunos também concordaram que conseguem expressar suas ideias e melhorar sua articulação e velocidade de fala. Além disso, eles afirmaram que conseguiam falar com mais fluência e precisão articulatória enquanto praticavam como locutores de rádio, leitores de notícias e repórteres profissionais, o que aumentou sua autoconfiança. Normalmente, as vozes consideradas mais adequadas para locução de rádio são aquelas de locutores profissionais e mais experientes^(36)^.

Destaca-se ainda o aumento significativo nas medidas CPP. Um resultado que sugere uma melhora na qualidade vocal em termos de clareza e proeminência das frequências de formantes^(37)^. O treinamento vocal pode aumentar o CPP ao melhorar a técnica vocal, reduzir ruídos indesejados, aprimorar a ressonância e a projeção vocal. Isso leva a uma produção vocal mais clara, distinta e de melhor qualidade, refletida em um CPP mais alto^(38,39)^. No entanto, é importante notar que não houve diferença significativa no CPPS, indicando que a suavidade da voz dos participantes permaneceu estável ao longo do treinamento^(40)^.

Houve uma diminuição da intensidade da fala após o treinamento e isso pode ser interpretado de diversas maneiras devido a diferentes fatores envolvidos no processo vocal. É importante considerar aspectos como a modulação e a expressividade vocal dos locutores ao analisar essa diminuição. Por exemplo, uma diminuição na intensidade da fala pode indicar uma mudança na técnica vocal dos locutores, resultando em uma produção vocal mais controlada e menos tensionada. Isso pode ser interpretado positivamente, indicando uma melhoria na qualidade vocal e uma redução na fadiga vocal^(12)^. No entanto, também é importante considerar a modulação e a expressividade vocal dos locutores. Uma diminuição na intensidade sem uma redução correspondente na expressividade vocal pode indicar uma perda de dinâmica na fala^(41)^, o que pode afetar a qualidade da comunicação. Por outro lado, se a diminuição na intensidade for acompanhada por uma modulação adequada e uma expressividade vocal consistente, isso pode indicar uma adaptação adequada da técnica vocal para diferentes contextos de comunicação^(8)^.

O programa demonstrou ser benéfico na melhoria da competência na comunicação dos locutores, o que tem implicações importantes para a formação de profissionais da voz falada em contextos radiofônicos. Esses resultados ressaltam a importância de programas de treinamento específicos e multidimensionais para o aprimoramento da competência na comunicação em locutores de rádio universitária.

Todavia, é importante mencionar algumas limitações deste estudo para que os resultados sejam analisados com cautela. Uma delas é a falta de um grupo de controle, o que torna difícil atribuir diretamente as mudanças observadas à intervenção, uma vez que outros fatores não controlados podem ou não influenciar nos resultados. Sobretudo, uma recente revisão da literatura científica sobre intervenções fonoaudiológicas com profissionais e estudantes de rádio e TV^(19)^ apontou que os delineamentos mais utilizados foram os estudos quase-experimentais e os estudos antes e após intervenção. Pelo fato das intervenções frequentemente ocorrerem em ambientes naturais e reais, como estúdios de rádio, onde é difícil estabelecer as condições de controle necessárias para um delineamento experimental completo. No entanto, este delineamento apresenta alto risco de viés e do efeito *Hawthorne*, um fenômeno em que os participantes alteram alguns de seus comportamentos por saberem que estão sendo estudados, independentemente da intervenção administrada.

O tamanho da amostra também merece atenção, pois uma amostra pequena pode limitar a capacidade de detectar efeitos significativos da intervenção^(19)^. A disponibilidade de estudantes que atuem em rádios para participar em pesquisas pode ser restrita, especialmente em áreas específicas ou em estudos que exigem tempo e comprometimento. Vale destacar que a atuação fonoaudiológica focada na competência na comunicação geralmente é realizada com pequenos grupos^(42)^. Estudos com grandes amostras e várias sessões podem exigir o recrutamento repetido de vários grupos ou a atuação simultânea de vários fonoaudiólogos, o que pode ser inviável, especialmente quando a intervenção é realizada no local de atuação desses participantes^(19)^.

Quanto à duração e intensidade do programa, é importante reconhecer que um programa de apenas duas horas ao longo de oito semanas pode ser considerado curto ou pouco intensivo para produzir mudanças significativas na comunicação oral dos locutores. Mas este parece ser um tempo comumente utilizado em intervenções fonoaudiológicas com estudantes que atuam em rádio^(19)^.

Além disso, a avaliação baseada apenas na autopercepção da fala e da voz, julgamento perceptivo-auditivo e análise acústica pode não abranger todos os aspectos relevantes da comunicação oral. Também deve ser considerada a possibilidade de efeitos de curto prazo, já que os resultados observados logo após a intervenção podem não se manter a longo prazo.

Por fim, é importante mencionar que não houve controle sobre variáveis externas, como mudanças na programação da rádio ou eventos externos. Além disso, não foram analisadas separadamente as diferenças entre homens e mulheres, que possuem configurações anatômicas e fisiológicas distintas que podem influenciar nas medidas acústicas. É essencial levar essas limitações em consideração ao interpretar e generalizar os resultados deste estudo.

## CONCLUSÃO

Os efeitos do Programa de Desenvolvimento da Expressividade para Comunicação Oral em locutores de rádio universitária indicam melhorias na competência na comunicação dos locutores com destaque para os resultados na autopercepção dos locutores em relação a dicção e ao som da voz. No julgamento perceptivo-auditivo das leituras, a metade dos pares avaliados mostrou melhorias após o treinamento, destacando o potencial do programa para o aprimoramento da qualidade da voz, na fala e na interpretação na leitura do texto informativo. Na análise acústica, houve mudanças na frequência fundamental da fala, taxa de articulação e elocução, sugerindo uma melhoria na qualidade vocal e na fluência da fala dos participantes. Embora não tenha havido diferença significativa na suavidade da voz, a diminuição da intensidade da fala pode ser interpretada como um aspecto da modulação e expressividade vocal.
